# Plasma membrane permeabilization following cell death: many ways to dye!

**DOI:** 10.1038/s41420-021-00545-6

**Published:** 2021-07-19

**Authors:** Elke De Schutter, Benjamin Cappe, Bartosz Wiernicki, Peter Vandenabeele, Franck B. Riquet

**Affiliations:** 1 VIB Center for Inflammation Research, Ghent, Belgium; 2 Department of Biomedical Molecular Biology, Ghent University, Ghent, Belgium; 3 Center of Medical Genetics, Antwerp University Hospital, University of Antwerp, Antwerp, Edegem Belgium; 4 Université de Lille, Lille, France

**Keywords:** Apoptosis, Assay systems, Membranes

In essence, apoptosis is a containment program preparing the cell corpse for engulfment by efferocytosis. When the phagocytic capacity is insufficient, apoptotic cells undergo disintegration accompanied by the release of cellular content, coined secondary necrosis. As secondary necrotic cells can elicit an inflammatory response [1], insights into the underlying mechanisms or molecules driving secondary necrosis are of major importance for therapeutic targeting. Rogers et al. reported that gasdermin E (GSDME), a member of the pore-forming gasdermin protein family and a substrate of the apoptotic caspase-3, drives secondary necrosis [2]. Indeed, *Gsdme*^−/−^ macrophages displayed lower plasma membrane permeabilization upon the stimulation with etoposide, an apoptosis inducer, as assessed by propidium iodide (PI) [2]. However, other reports showed that the loss of GSDME did not result in differential kinetics in plasma membrane permeabilization measured by TO-PRO-3 fluorescent DNA dye in UV irradiated THP-1 and Jurkat T cells [3]. Similarly, the absence of GSDME did not affect membrane permeabilization measured by YOYO-1 fluorescent DNA dye in macrophages upon anti-Fas treatment [4].

In theory, impermeant DNA-binding dyes seem ideal for assessing the membrane permeabilizing function of GSDME. These dyes enter and bind to nuclear DNA and allow detection of cells that lose membrane integrity. Most of them demonstrate little to no solution fluorescence and greatly increased quantum yield when bound to DNA [5–7]. However, most of these dyes were initially designed to stain DNA before being repurposed to stain cells with compromised membranes. Although their binding and membrane passing characteristics are largely unknown, all of these cell impermeant nuclear dyes are used to measure plasma membrane permeabilization. Importantly, some of these small cationic nuclear dyes were shown to enter cells by pannexin channels mediated mechanisms (YO-PRO-1, and TO-PRO-3) [8, 9], allowing for labeling early apoptotic cells [10, 11] associated with the activation of pannexin-1 and making their applicability in measuring plasma membrane permeabilization problematic.

In that respect, the contradicting results concerning the role of GSDME during apoptosis-driven secondary necrosis might be attributed to the choice of nucleic acid stain to measure plasma membrane permeabilization rather than to differences in cellular context or GSDME expression levels. Next to PI and 7-AAD, SYTOX dyes are commonly used as dead-cell markers as they provide some advantages to other dyes, such as a good signal-to-noise ratio at low concentrations and a low photobleaching rate [6, 12]. Despite the small size of SYTOX cyanine nuclear DNA dyes (<0.6 kDa) they are not associated with pannexin-mediated mechanisms nor known to label early apoptotic cells.

In the light of these conflicting findings, we decided to investigate GSDME function in the murine fibrosarcoma cell line L929sAhFas, susceptible to apoptosis when stimulated with human Fas antibody [13], and generated L929sAhFas GSDME KO cell lines (Fig. 1A). Surprisingly, using 7-AAD and SYTOX blue (SB) dyes combined, we came across a differential staining pattern in the absence of GSDME expression in L929sAhFas during apoptosis-driven secondary necrosis. Upon anti-Fas treatment, L929sAhFas wild-type clones proceeded from a double negative (7-AAD−/SB−) population toward a double positive population (7-AAD+/SB+) (Fig. 1B, C), while in L929sAhFas *Gsdme-*deficient clones, a clear 7-AAD single positive stage (7-AAD+/SB−) (Fig. 1B) and a decrease in SB uptake (Fig. 1D) was observed. Doxycycline-inducible mGSDME reconstitution and anti-Fas stimulation in one L929sAhFas *Gsdme-*deficient clone (Fig. 1E) rescued the reduced SB staining (Fig. 1F, G) in absence of GSDME expression into the simultaneous 7-AAD and SB staining (Fig. 1F, H) initially observed in L929sAhFas wild type (Fig. 1B, C). This suggests that the observed distinct uptake patterns were due to the presence and absence of GSDME. Live cell imaging confirmed a delay in nuclear staining by SB compared to 7-AAD in cells lacking GSDME (Fig. 1J, K, Supplementary videos). Interestingly, GSDME expression had no effect on the uptake of 7-AAD in our analysis, suggesting that other, GSDME independent, cell membrane permeabilization mechanisms could operate in L929sAhFas allowing uptake 7-AAD upon anti-Fas treatment and excluding SB uptake. As reconstitution of GSDME expression resulted in the simultaneous uptake of 7-AAD and SB upon anti-Fas treatment (Fig. 1I, L), our results suggest that GSDME favors cell membrane permeabilization mechanisms allowing nuclear staining by SB, while 7-AAD is entering the cell by another mechanism.

**Fig. 1:**
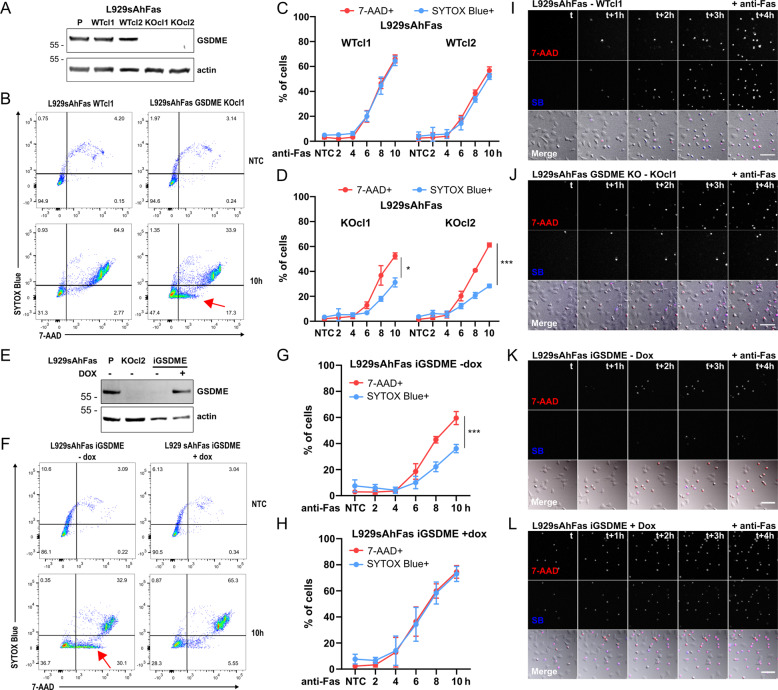
Manipulation of GSDME affects 7-AAD and SB cell death marker dyes pattern in L929sAhFas during apoptosis-driven secondary necrosis. **A** Western Blot showing GSDME protein expression in L929sAhFas clones modified with CRISPR-Cas9 gene editing. **B–D** Flow cytometry analysis of the uptake of SB and 7-AAD in L929sAhFas WT and GSDME KO clones upon anti-Fas treatment. Results are presented as means ± SD (*N* = 3). **B** Representative flow cytometric plots with red arrows pointing to 7-AAD single positive cells. **C** Levels of 7-AAD (red) and SB (blue) positive cells given as percentage of total cell population upon anti-Fas treatment for two L929sAhFas GSDME WT clones. **D** Percentage of 7-AAD and SB positive cells given as percentage of total cell population upon anti-Fas treatment for two GSDME KO clones. **E** Western Blot showing doxycycline-dependent expression of GSDME in L929sAhFas iGSDME cells compared to GSDME expression in the L929sAhFas parental (P) cells and in L929sAhFas GSDME KOcl2. **F–H** Flow cytometry analysis of the SB and 7-AAD staining in L929sAhFas iGSDME with or without doxycycline pretreatment upon apoptosis induction by anti-Fas. Results are presented as means ± SD (*N* = 5). **F** Representative flow cytometry plots. **G** Percentage of 7-AAD and SB positive L929sAhFas iGSDME cells without doxycycline pretreatment upon anti-Fas treatment. **H** Effect of GSDME expression due to treatment with doxycycline on the amount of 7-AAD and and SB positive L929sAhFas iGSDME cells upon anti-Fas treatment. **I–L** Confocal images of L929sAhFas WT, GSDME KO cl1, iGSDME (− Dox) and iGSDME (+ Dox) cells upon anti-Fas treatment showing uptake of 7-AAD and SB over time. h hours, dox doxycycline, 7-AAD 7-aminoactinomycin D, SB SYTOX blue; scale bar = 100 µM. Videos available in supplementary material.

Thus, our results based on SB staining are consistent with the findings of Rogers et al. reporting that GSDME is responsible for membrane permeabilization during apoptosis-driven secondary necrosis [2]. On the opposite, our results based on 7-AAD staining support the findings of Tixeira et al. and Lee et al. stating that GSDME does not affect cell membrane permeabilization during apoptosis-driven secondary necrosis [3, 4]. Therefore, caution is needed when drawing conclusions from different studies using various cell death markers. Additional studies using markers of late apoptotic cells could clarify the current contradicting findings and assess the importance of the choice of marker to investigate cell membrane permeabilization mechanisms. Altogether, it seems that next to pannexin channels mediated mechanism, different GSDME dependent and independent mechanisms leading to membrane permeabilization take place during apoptosis-driven secondary necrosis, allowing the selective uptake of different nuclear dyes. Although the use of cell impermeant dyes might not be suitable to study membrane permeabilization processes itself, the double staining with SB and 7-AAD may allow functionality studies of GSDME and maybe other GSDMs.

## Supplementary Information

Supplementary material
